# *Escherichia coli*-derived recombinant human bone morphogenetic protein-2 combined with bone marrow-derived mesenchymal stromal cells improves bone regeneration in canine segmental ulnar defects

**DOI:** 10.1186/s12917-016-0829-y

**Published:** 2016-09-13

**Authors:** Takamasa Itoi, Yasuji Harada, Hiroyuki Irie, Michiko Sakamoto, Katsutoshi Tamura, Takuya Yogo, Satoshi Soeta, Hajime Amasaki, Yasushi Hara, Masahiro Tagawa

**Affiliations:** 1 Division of Veterinary Surgery, Nippon Veterinary and Life Science University, 1-7-1 Kyonan-cho, Musashino, Tokyo 180-8602 Japan; 2 HOYA Technosurgical Corporation, 1-1-110 Tsutsujigaoka, Akishima, Tokyo 196-0012 Japan; 3 Division of Animal and Clinical Regenerative Medicine, Kurashiki University of Science and Arts, 2640 Nishinoura, Tsurajima-machi, Kurashiki, Okayama 712-8505 Japan; 4 Division of Veterinary Anatomy, Nippon Veterinary and Life Science University, 1-7-1 Kyonan-cho, Musashino, Tokyo 180-8602 Japan

**Keywords:** Recombinant human bone morphogenetic protein-2, Bone marrow-derived mesenchymal stromal cells, Bone regeneration, Canine, Ulnar defect

## Abstract

**Background:**

Large bone defects in canines usually require assistance to achieve healing. Implantation of osteoinductive factors can promote bone healing, while transplantation of osteoprogenitor cells can enhance bone regeneration. We hypothesized that implantation of an osteoinductive factor, recombinant human bone morphogenetic protein-2 (rhBMP-2), combined with osteoprogenitor cells, bone marrow-derived mesenchymal stromal cells (BMSCs), would synergistically promote bone healing. In this study, we examined the combined effects of *Escherichia coli*-derived rhBMP-2 and BMSCs on bone healing after implantation into canine ulnar defects.

**Results:**

Critical-sized osteoperiosteal segmental defects (2.5 cm) were created in the ulnae of healthy female beagle dogs, and implanted with combinations of *E. coli*-derived rhBMP-2 (560 or 140 μg) and autologous BMSCs (10^7^, 10^5^, or 0 cells). In the present study,18 forelimbs of nine healthy purpose-bred female beagles were used. All six treatment groups contained three forelimbs, and the animals were euthanized after 12 weeks. The control groups (560 and 140 μg/0 cells) were cited from our previous study to reduce the number of experimental animals. Radiographically, the regenerated bone width was significantly increased in the 560 or 140 μg with 10^7^ and 10^5^ cells groups compared with the 0 cells groups. By quantitative CT, the bone mineral density was higher in the 560 μg with 10^7^ and 10^5^ cells groups, while non-uniformity of the bone mineral density was improved in the 560 μg with 10^7^ and 10^5^ cells groups and 140 μg/10^7^ cells group. Mechanically, the maximum loads at failure were significantly higher in the 560 μg with 10^7^ and 10^5^ cells groups. Histologically, the regenerated bone was well-developed and contained osteocyte-like cells marrow cavities, and vessels. However, the osteoclasts and osteoblasts were hardly observed. The osteocyte-like cell numbers were significantly higher in the 560 μg with 10^7^ and 10^5^ cells and 140 μg with 10^7^ and 10^5^ cells groups.

**Conclusions:**

Implantation of *E. coli*-derived rhBMP-2 and BMSCs led to significantly enhanced bone formation, with improved bone mineral density and reduced non-uniformity of the regenerated bone. Combined implantation of rhBMP-2 and BMSCs may be useful for promotion of bone healing in critical-sized defects in canines.

## Background

Many studies have suggested that osteoinductive factors, such as bone morphogenetic proteins (BMPs), can greatly increase bone regeneration [1–7]. Currently, recombinant human bone morphogenetic protein-2 (rhBMP-2) is used in veterinary medicine for treatment of canines with non-unions [8, 9], arthrodesis [10, 11], and mandibular defects [12, 13].

In a previous study, we evaluated the healing efficacy of *Escherichia coli*-derived rhBMP-2 in a canine model [14]. The animals administered higher doses of rhBMP-2 (560 μg/site or more) showed larger callus formation and integration with the host bone, while those administered lower doses (140 μg/site or less) showed smaller callus formation and incomplete integration the host bone. However, the higher doses of rhBMP-2 also led to transient decreases in bone mineral density during the early stage of treatment. Because rhBMP-2 promotes bone regeneration by activating progenitor cell recruitment, proliferation, and differentiation [15–19], we hypothesized that implantation of rhBMP-2 combined with osteoprogenitor cells would improve bone regeneration.

Bone marrow-derived mesenchymal stromal cells (BMSCs) comprise a heterogeneous population of multipotent cells, including mesenchymal stem cells, which have frequently been investigated as a cell source for regenerative medicine applications [20–23]. Mesenchymal stem cells were first isolated from bone marrow in 1991 by Caplan [24], and have the potential to differentiate into multilineage cells, including osteoblasts. Several studies have examined the regeneration of bone defects following transplantation of BMSCs combined with artificial bone ceramics [25, 26]. Thus, we hypothesized that the combination of *E. coli*-derived rhBMP-2 and BMSCs would synergistically affect bone regeneration. The purpose of this study was to determine the effects of implantation of rhBMP-2 combined with BMSCs on the regeneration of segmental canine ulnar defects. The newly formed bone structures and mechanical strengths were evaluated by X-rays, computed tomography (CT), mechanical testing, and histology.

## Methods

### Experimental design

Because this study was performed using identical conditions to our previous study [14] and we wanted to reduce the number of experimental animals, the data for the two control groups not implanted with cells are cited from our previous study.

In the study, 18 forelimbs of nine healthy purpose-bred female beagle dogs (age: 1 year; body weight: 9.4–11.0 kg) were used. The animals were maintained under uniform conditions in our institutional animal laboratory. The surgical techniques were described previously [14]. Briefly, at 15 min after administration of droperidol (0.25 mg/kg, intramuscular; Sankyo Co., Tokyo, Japan), anesthesia was induced with propofol (7 mg/kg, intravenous; Fuji Pharma Co. Ltd., Toyama, Japan) and maintained with inhalation of isoflurane (1.5–2.0 %; Mylan Inc., Osaka, Japan) and oxygen. Before surgery, 2 % lidocaine hydrochloride (1 mL/4.5 kg; AstraZeneca, Osaka, Japan) and 0.5 % bupivacaine hydrochloride (1 mL/4.5 kg; AstraZeneca) were injected around the brachial plexus to induce local anesthesia. Critical-sized osteoperiosteal segmental defects of 2.5 cm were created on the ulnae of the canines distal to the interosseous ligament using an oscillating saw. The defects were approximately three times larger than the diameter of the ulnar shaft at its mid-point, reported as a critical size that does not heal spontaneously [27].

After defect creation, 700 mg of β-tricalcium phosphate granules containing *E. coli*-derived rhBMP-2 and autologous BMSCs was implanted into the defect. The experimental forelimbs were divided into six groups of three forelimbs treated with 560 or 140 μg of rhBMP-2 and 10^7^, 10^5^, or 0 BMSCs, designated 560 μg/10^7^ cells, 560 μg/10^5^ cells, 560 μg/0 cells, 140 μg/10^7^ cells, 140 μg/10^5^ cells, and 140 μg/0 cells. The perioperative analgesic management included preoperative and postoperative administration of buprenorphine (20 μg/kg, intramuscular; Otsuka Pharmaceutical Co. Ltd., Tokyo, Japan). Buprenorphine was administered twice a day for 3 days postoperatively. For 7 days postoperatively, 25 mg/kg ampicillin was administered orally twice daily. The forelimbs underwent radiographic examinations preoperatively and postoperatively, and then weekly thereafter using a lateral view, and the width of the regenerated bone was measured. CT scanning was performed with 1.0-mm slice thickness in the axial plane at time 0 (post-operation) and at 4, 8, and 12 weeks postoperatively with the canines under general anesthesia. At 12 weeks postoperatively, the animals were euthanized with an overdose of injected pentobarbital sodium (Somnopentyl; Kyoritsu Seiyaku Co., Tokyo, Japan). The tissues, including the regenerated bones, were harvested for mechanical and histological examinations. As noted above, the data for the two control groups not treated with cells are cited from our previous study [14], and thus mechanical testing was not performed on these two groups.

### Preparation of the implants

The *E. coli*-derived rhBMP-2 used in this study was supplied by Osteopharma Inc. (Osaka, Japan). Although *E. coli*-derived rhBMP-2 is not glycosylated, unlike Chinese hamster ovary cell-derived rhBMP-2, a previous in vitro study suggested that the biological activities of rhBMP-2 derived from these two different cellular origins are similar [28]. The biodegradable artificial bone β-tricalcium phosphate granules used were produced by HOYA Technosurgical Corporation (Tokyo, Japan). The granules had diameters of 2–4 mm, and were composed of interconnecting porous structures (pore size: 50–300 μm; porosity: 75 %). Freeze-dried *E. coli*-derived rhBMP-2 powder (140 or 560 μg) was reconstituted in 0.32 mL of sterile distilled water before use. Approximately 15 min before implantation, 0.32 mL of rhBMP-2 solution was dripped onto 700 mg of β-tricalcium phosphate granules and allowed to infiltrate.

Autologous BMSCs were isolated from the bone marrow of each animal at 10 days before transplantation. Under general anesthesia, bone marrow was aspirated from the proximal humerus using standard bone marrow biopsy techniques. A sterile 13-gauge Jamshidi needle was used to aspirate 5 mL of bone marrow into a syringe containing 5 mL of heparinized (1,000 U/mL) saline solution. The techniques reported by Kadiyala et al. [29] were used for bone marrow harvesting and BMSC isolation. To obtain BMSC-enriched nucleated cells, density separation was performed using Lymphoprep (Axis-Shield, Oslo, Norway). Briefly, bone marrow cells in phosphate-buffered saline (10 mL) were carefully layered onto 5 mL of Lymphoprep, and separated by centrifugation at 800 × *g* for 30 min at room temperature. The nucleated cells were collected from the solution/Lymphoprep interface, washed in phosphate-buffered saline, and plated in T-75 cell culture flasks with 10 mL of Dulbecco’s modified Eagle’s medium supplemented with 10 % fetal bovine serum and 1 % antibiotic/antimycotic solution (control medium). The cells were incubated at 37 °C in a humidified 5 % CO_2_ environment, and the medium was changed twice weekly. When the primary cultures reached 70–80 % confluency, the attached cells were passaged by exposure to 0.25 % trypsin/1 mM EDTA for 3 min. The detached cells were replated at 8.0 × 10^3^ cells/cm^2^. Either 10^7^ or 10^5^ BMSCs were suspended in 0.32 mL of saline and seeded onto the rhBMP-2-treated β-tricalcium phosphate granules immediately before implantation. Saline alone (0.32 mL) was seeded onto the rhBMP-2-treated β-tricalcium phosphate granules in the groups receiving 0 cells.

### Width of the regenerated bone

**Fig. 1:**
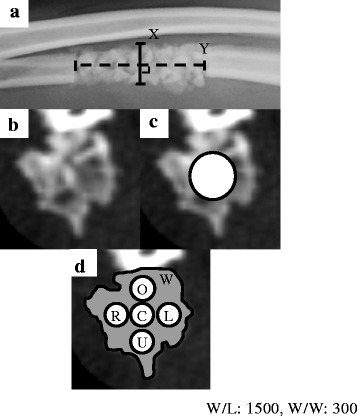
Measurement methods for the experiments. **a** Lateral-view X-ray of the experimental region. The width of the regenerated bone was determined by calculating the length of the perpendicular line “X” at the midpoint of the longitudinal line “Y” in the defect. **b** Transverse-plane CT image of the central portion of the regenerated bone. **c** The bone mineral density was calculated by measuring the CT-determined Hounsfield units in the 5.5-mm diameter ROI at the center of the regenerated bone and the calibration phantom. **d** The non-uniformity of the bone mineral density was calculated by measuring the CT-determined Hounsfield units in the whole area W and in five points within the specified 1.5-mm diameter ROI

The width of the regenerated bone was measured on a lateral view radiograph. An aluminum plate (25 mm × 74 mm) was exposed alongside the forelimbs to standardize the magnification and contrast for all X-ray images. A line perpendicular to the midpoint of the long axis of the regenerated bone was measured, and the distance between the outer edges of the intersections of the line was defined as the regenerated bone width (Fig. 1a).

### Bone mineral density of the regenerated bone

The bone mineral density of the regenerated bone was measured using a quantitative CT method [30, 31] on a transverse plane reconstruction in the middle region of the defect. Quantitative CT is a volumetric method for estimating the mass of hydroxyapatite per cubic centimeter of bone tissue. The slice thickness was maintained at 1 mm, and the forelimbs were scanned along with a calibration phantom (B-MAS200; Kyoto Kagaku Co. Ltd., Kyoto, Japan). A multiplanar reconstruction mode was used for the analysis (Fig. 1b). Twenty-five slices of 1-mm thickness were reconstructed in each defect with correct alignment, and the thirteenth slice was defined as the center slice for assessment. The window width and window level were set at 1500 and 300 Hounsfield units, respectively. The mean Hounsfield units in a specified 5.5-mm diameter region of interest (ROI) were determined at the center of the regenerated bone and for the calibration phantom, after which the mean value of the bone mineral density was calculated (Fig. 1c).

### Non-uniformity of the bone mineral density

The non-uniformity of the bone mineral density was calculated in the same CT image used to measure the bone mineral density. The mean bone mineral density for the whole area (W) and the mean bone mineral density inside a specified 1.5-mm diameter ROI based on five points were measured (Fig. 1d). The non-uniformity index was calculated as the root-mean-square deviation of the five measurement points, with W used as the “true” average. A completely uniform field resulted in a non-uniformity index of 0, and higher values indicated increased non-uniformity.

### Mechanical testing

As noted above, the data for the two control groups not treated with cells are cited from our previous study [14], and thus mechanical testing was not performed on these two groups. After euthanasia of the animals at 12 weeks, the tissues including the regenerated bones were harvested with extra margins as necessary for mechanical testing. A three-point bending test was performed using a universal testing machine (858Mini Bionix II; MTS Systems Corporation, Tokyo, Japan). The crosshead speed was 2.5 mm/min, the distance between the fulcrum points was 60 mm, and the load was applied from the cranial side. The tests were destructive to allow determination of the maximum load at breaking point.

### Histology

Immediately after the mechanical testing, the samples were fixed in 4 % paraformaldehyde for 2 days and then decalcified with EDTA. The decalcified samples were embedded in paraffin and sectioned in the sagittal plane at 3-μm thickness. The sections were stained with hematoxylin and eosin to show the structure of the regenerated bone. The density of osteocyte-like cells was calculated by counting the cell number and determining the area of newly formed trabecular bone within each field of view.

### Statistical analysis

A two-way repeated measures ANOVA was used to investigate the effects of the different transplanted BMSC numbers with 560 μg or 140 μg of rhBMP-2 on the width of the regenerated bone, bone mineral density of the regenerated bone and non-uniformity of the bone mineral density. When a main effect was found, Tukey’s honest significant difference test was used to compare the mean values between the groups at each time point.

A one-way repeated measures ANOVA was used to investigate the effects of the different transplanted BMSC numbers with 560 μg or 140 μg of rhBMP-2 on the mechanical testing and density of osteocyte-like cells. When a significant difference was found, Tukey’s honest significant difference test was used to compare the mean values between the groups.

Values of *P* < 0.05 were considered statistically significant. All statistical analyses were performed using IBM SPSS Statistics, Version 16.0 (IBM Corp., Tokyo, Japan). All data are presented as mean ± SD.

## Results

The surgery was performed smoothly with no postoperative pain in all groups. No complications developed in any of the groups during the study period.

### X-ray analysis

**Fig. 2:**
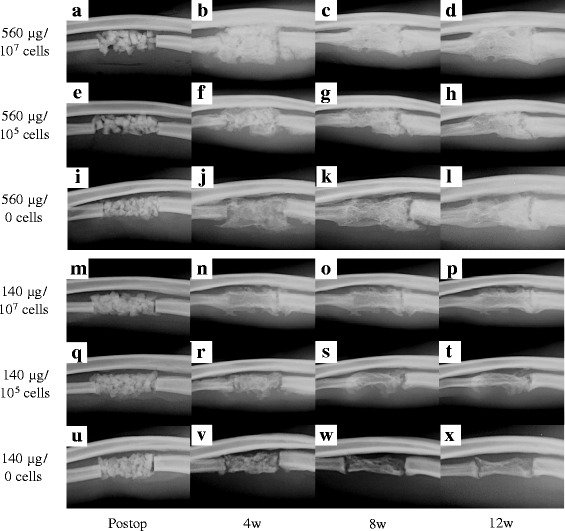
Radiographs of the postoperative changes. **a**–**x** Postoperative images taken at 4, 8 and 12 weeks after surgery are shown for all six experimental groups: 560 μg rhBMP-2 with 10^7^ (**a**–**d**), 10^5^ (**e**–**h**), and 0 (**i**–**l**) cells, and 140 μg rhBMP-2 with 10^7^ (**m**–**p**), 10^5^ (**q**–**t**), and 0 (**u**–**x**) cells. The groups transplanted with BMSCs form more regenerated bone than the groups without cells at the same dose of rhBMP-2

Radiographs highlighting the postoperative changes in the six groups are shown in Fig. 2. At 4 weeks after surgery, the callus expansion peaked in all groups treated with 560 μg of rhBMP-2 and in the 140 μg/10^7^ cells group, and the border between the regenerated bone and the host bone was nearly unidentifiable, especially in the distal region. In contrast, the 140 μg/10^5^ cells group and one sample in the 140 μg/0 cells group showed relatively small callus formation. No external callus formation was observed in the other two samples in the 140 μg/0 cells group. A gap was visible between the graft and the host bone in the groups treated with 140 μg rhBMP-2 and 10^5^ or 0 cells. The granularity of the implanted materials disappeared in all samples in all groups.

At 8 weeks after surgery, the remodeling process had progressed in all three groups treated with 560 μg rhBMP-2 and in the 140 μg/10^7^ cells group, and the outline of the regenerated bone had adapted to fit the shape of the host bone, particularly on the distal side. In contrast, no changes were visible in the outline of the regenerated bone in the 140 μg groups with 10^5^ and 0 cells. The distal side of the regenerated bone was completely integrated with the host bone in all three groups treated with 560 μg rhBMP-2 and in the 140 μg/10^7^ cells group. On the proximal side, the regenerated bone was integrated for one sample in the 560 μg/10^7^ cells group and two samples in the 560 μg/10^5^ cells group. A radiolucent gap was observed at the proximal side for all samples in the 560 μg/0 cells group, but the cells were bridged to the host bone. Two samples in the 140 μg/10^5^ cells group showed integration at the distal end, but all samples in the 140 μg/0 cells group showed a radiolucent gap on each side.

At 12 weeks after surgery, the distal part of the regenerated bone was completely integrated with the host bone in all samples in the three groups treated with 560 μg rhBMP-2 and in the 140 μg rhBMP-2 with 10^7^ and 10^5^ cells groups. The proximal part of the regenerated bone was also integrated with the host bone in all samples in the 560 μg with 10^7^ and 10^5^ cells groups, as well as in the 140 μg/10^7^ cells group. Although a small gap was present at the proximal side of the lesion, it was covered by a callus in all samples in the 560 μg/0 cells group. The width of the regenerated bone was larger than or equal to that of the proximal ulna in all samples in the three groups treated with 560 μg and in the 140 μg with 10^7^ and 10^5^ cells groups. In two samples in the 140 μg/0 cells group, the regenerated bone was not integrated with the host bone and a distinct gap remained on each side. The width of the regenerated bone was nearly equal to or less than that of the proximal ulna.

### Changes in the width of the regenerated bone over time

**Fig. 3:**
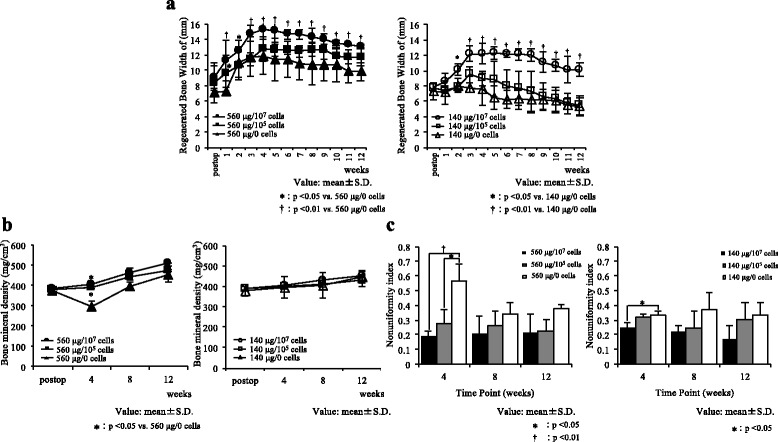
X-ray examination. (Radiohraphic and CT examination). **a** Sequential changes in the width of the regenerated bone over time. The changes in the width of the regenerated bone (mean ± SD) over time are shown. The width of the regenerated bone is significantly larger in the groups treated with BMSCs than that in the groups treated with rhBMP-2 alone. *P < 0.05 vs 560 μg/0 cells or vs. 140 μg/0 cells. †*P* < 0.01 vs. 560 μg/0 cells or vs. 140 μg/0 cells. **b** Changes in the bone mineral density over time. The changes in the regenerated bone mineral density (mean ± SD) over time are shown. The inclusion of 10^7^ and 10^5^ BMSCs is able to prevent the transient decrease in the bone mineral density observed at 4 weeks after surgery for the high dose of rhBMP-2 without cells. The regenerated bone mineral densities in the 560 μg with 10^7^ and 10^5^ cells groups are significantly larger than that in the 560 μg/0 cells group at 4 weeks. No significant differences in the bone mineral density are found among the groups treated with 140 μg of rhBMP-2. *P < 0.05 vs. 560 μg/0 cells. **c** Non-uniformity index values of the bone mineral density in the regenerated bone. The changes in the non-uniformity index of the regenerated bone mineral density (mean ± SD) over time are shown. The non-uniformity index in the groups transplanted with both BMSCs and rhBMP-2 are significantly lower than those in the groups treated with rhBMP-2 alone at 4 weeks. The new bone in the 560 μg with 10^7^ and 10^5^ cells groups and in the 140 μg with 10^7^ and 10^5^ cells groups is more uniform than that in their respective control groups with 0 cells. *P < 0.05; †*P* < 0.01

The sequential changes in the width of the regenerated bone over time after surgery measured on a lateral radiograph are shown in Fig. 3a. In the 560 μg/10^7^ cells group, the width of the regenerated bone increased rapidly, peaked at 4 weeks, and then decreased slowly until 12 weeks. In the 560 μg/10^5^ cells group, the width of the regenerated bone increased during the first 4 weeks, reached a peak, and then remained at the same level until 12 weeks. In the 560 μg/0 cells group, the regenerated bone width increased rapidly for 2 weeks, peaked at 4 weeks, and decreased slowly thereafter until 12 weeks. The widths of the regenerated bone in the 560 μg/10^7^ cells group were significantly larger than those in the 560 μg/0 cells group at 1 to 12 weeks after transplantation. Furthermore, the width of the regenerated bone in the 560 μg/10^5^ cells group was significantly larger than that in the 560 μg/0 cells group at 1 week after transplantation.

In the 140 μg/10^7^ cells group, the regenerated bone width increased rapidly, peaked at 5 weeks, and then slowly decreased until 12 weeks. In the 140 μg/10^5^ cells group, the regenerated bone width increased slowly, reached a peak at 3 weeks, and slowly decreased thereafter until 12 weeks. In the 140 μg/0 cells group, the regenerated bone width increased slightly, peaked at 2 weeks, and then decreased slowly until 12 weeks. The regenerated bone widths in the 140 μg/10^7^ cells group were significantly larger than those in the 140 μg /0 cells group at 2 to 12 weeks after transplantation. Significant differences were found between the regenerated bone widths in the 140 μg/10^7^ cells and 560 μg/10^7^ cells groups, but not between the 140 μg/10^7^ cells and 560 μg/10^5^ cells groups.

### Changes in the bone mineral density of the regenerated bone over time

The changes in the bone mineral density over time after surgery, as measured by a quantitative CT method, are shown in Fig. 3b. The bone mineral densities of the implanted materials assessed postoperatively were nearly equal between- group. The bone mineral density in the 560 μg with 10^7^ and 10^5^ cells groups was slightly increased at 4 weeks and continued to increase until 12 weeks. The bone mineral density in the 560 μg/0 cells group showed a transient decrease at 4 weeks, but then increased until 12 weeks. The bone mineral density in the 560 μg/0 cells group was significantly lower than those in the 560 μg with 10^7^ and 10^5^ cells groups at 4 weeks. The bone mineral density in all three groups treated with 140 μg rhBMP-2 showed similar tendencies with no significant differences between the groups.

### Non-uniformity of the bone mineral density

The non-uniformity index values for the bone mineral density of the regenerated bone after surgery in the different groups, as measured by the quantitative CT method, are shown in Fig. 3c. The non-uniformity index was significantly lower in the 560 μg with 10^7^ and 10^5^ cells groups than in the 560 μg/0 cells group at 4 weeks. Similarly, the non-uniformity index in the 140 μg/10^7^ cells group was significantly lower than that in the 140 μg/0 cells group at 4 weeks.

### Mechanical testing

**Fig. 4:**
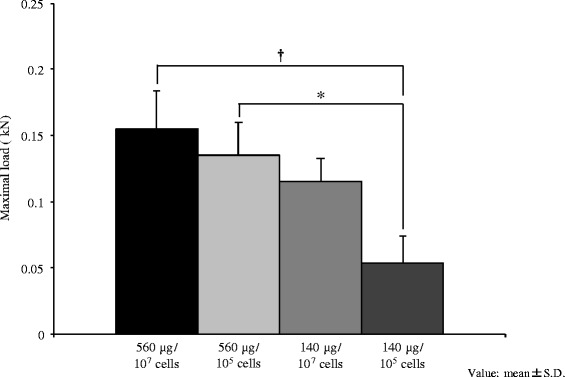
Mechanical testing. This experiment was carried out in the 560 μg with 10^7^ and 10^5^ cells groups and the 140 μg with 10^7^ and 10^5^ cells groups, because the 560 μg/0 cells group and 560 μg/0 cells group are cited from our previous study The maximum loads at failure of the regenerated bone using a three-point bending test (mean ± SD) are shown. The regenerated bone in the 560 μg with 10^7^ and 10^5^ cells groups is mechanically stronger than that in the 140 μg/10^5^ cells group. *P < 0.05; †*P* < 0.01

The results of the mechanical testing are shown in Fig. 4. The maximum loads at failure in the 560 μg with 10^7^ and 10^5^ cells groups were significantly higher than that in the 140 μg/10^5^ cells group.

### Histology

**Fig. 5:**
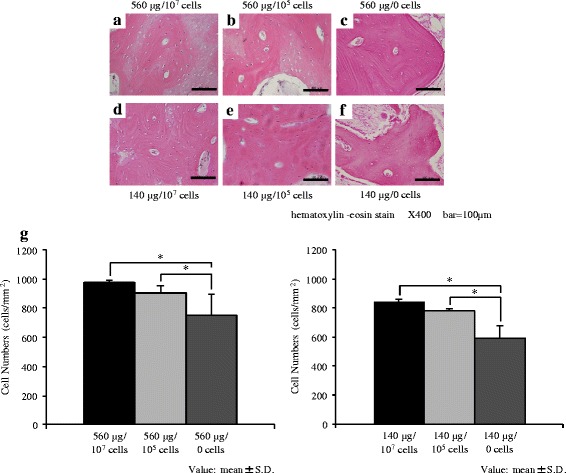
Histology of the regenerated bone. **a**–**f** Representative histological sections of the regenerated bone at 12 weeks after surgery stained with hematoxylin and eosin (×400, scalebar = 100 μm). The groups treated with 560 μg rhBMP-2 with 10^7^ (**a**), 10^5^ (**b**), and 0 (**c**) cells, and 140 μg rhBMP-2 with 10^7^ (**d**), 10^5^ (**e**), and 0 (**f**) cells are shown. Scale bar: 500 μm. **g** Numbers of osteocyte-like cells per trabecular bone area. The numbers of osteocyte-like cells in the trabecular bone (mean ± SD) are shown. The numbers of osteocyte-like cells are significantly increased by transplantation with BMSCs. *P < 0.05

Representative images for the histology of the regenerated bone (magnification; ×400) are shown in Fig. 5a. The newly formed trabecular bone was well developed and contained osteocyte-like cells, marrow cavities, and vessels. The structure and thickness of the trabecular bone were similar among the different groups. In all samples, many flattened of spindle cells were observed, while osteoblasts that were activated and cuboidal were hardly observed. Osteoclasts that were multinuclear with large sizes were also hardly observed.

The numbers of osteocyte-like cells per trabecular bone area in the different groups are shown in Fig. 5b. The 560 μg with 10^7^ and 10^5^ cells groups had significantly more osteocyte-like cells than the 560 μg/0 cells group. Similarly, the numbers of osteocyte-like cells were significantly larger in the 140 μg with 10^7^ and 10^5^ cells groups than in the 140 μg/0 cells group.

## Discussion

The results of the present study show that implantation of rhBMP-2 combined with BMSCs led to significantly larger amounts of bone formation than implantation of rhBMP-2 alone. The mechanism of action for the effects of BMPs involves regulation of gene expression by the transfer of bone-inducing signals, and induction of new bone formation by activating the proliferation and differentiation of mesenchymal stem cells into osteoblasts [15]. The results of the present study suggest that BMSCs were activated by rhBMP-2 following implantation BMSCs with rhBMP-2, thus promoting bone formation.

Meanwhile, although rhBMP-2 was reported to show an effect on bone formation induction in a dose-dependency manner [32], the promotion of bone resorption with its high-dose use is of concern. This phenomenon is thought to arise because BMP signaling is involved not only in osteoblast differentiation, but also in regulating osteoclast formation. Despite the fact that osteoclasts are formed by hematopoietic progenitors of the monocytic lineage, proper BMP-dependent maturation of osteoblasts is required for osteoclast differentiation to occur [33]. Seeherman et al. [34] reported rhBMP-2-induced transient bone resorption prior to bone formation after implantation of rhBMP-2 with an absorbable sponge in the metaphyseal region of femurs. Bone resorption around the defect was observed radiologically by 2 weeks after surgery, and histologically at 1 week after surgery. In the present study, the 560 μg/0 cells group showed a transient decrease in the bone mineral density at 4 weeks after surgery. This finding suggests that the high dose of rhBMP-2 increased bone resorption during the early phase of bone regeneration. However, the 560 μg rhBMP-2 with 10^7^ and 10^5^ cells groups did not show any transient decreases in the bone mineral density. In addition, the non-uniformity index in the 560 μg/10^7^ cells group was significantly lower than that in the 560 μg/0 cells group at 4 weeks after surgery, and remained at the same low level for the rest of the experiment. These findings suggest that transplantation should be performed with BMSCs to inhibit *E. coli*-derived rhBMP-2-induced bone resorption.

Because the bone-inducing ability of rhBMP-2 is dose-dependent [32], BMSCs were transplanted at 10^7^ and 10^5^ cells in the present study. The regenerated bone width in the 140 μg/10^7^ cells group increased to nearly the same level as those in the 560 μg rhBMP-2 with 10^5^ and 0 cells groups. Moreover, the mechanical strength in the 140 μg/10^7^ cells group did not differ significantly from those in the 560 μg with 10^7^ and 10^5^ cells groups. In addition, although the data are not shown, the maximum load at failure in the 560 μg/10^7^ cells group did not differ significantly from that for intact canine ulnae. These findings suggest that BMSCs also had an effect according to the number of the transplanted cells.

The regenerated bone in the present study was confirmed to show a definite bone structure by histological examination. However, osteoclasts and osteoblasts, which are important for bone remodeling, were hardly observed. TGF-β promotes osteoclast apoptosis [35], and apoptotic osteoclasts are quickly removed by phagocytes [36]. Furthermore, rhBMP-2 promotes the differentiattion of BMSCs into osteoblasts [15]. Meanwhile, in their final phase of differentiation, osteoblasts become osteocytes [37]. In the present study, the histological examination was performed at only 12 weeks after transplantation once the experimental period had ended. Based on the above observations, it is suggested that osteoclasts and osteoblasts were not observed, though many osteocytes were observed. The number of osteocytes is an important factor that determines bone mass and strength [38] In the present study, the numbers of osteocyte-like cells in the histological examinations confirmed the results of the X-ray examination and mechanical testing.

Sciadini et al. [9] reported that bone-void structures, which may occur when the carrier is absorbed before being replaced by new bone, were present when high doses of rhBMP-2 were used. Seeherman et al. [34] also reported the appearance of transient bone resorption prior to bone formation in the X-ray and histological examinations. In the present study, bone-void structures were mostly found in the 560 μg/0 cells group, which also showed the highest non-uniformity index among all the experimental groups. However, the bone-void number and non-uniformity index of the bone mineral density appeared to be improved in both the 560 μg with 10^7^ and 10^5^ cells groups, although the bone-void numbers did not differ significantly between the groups (data not shown). Further studies are needed to determine the mechanism underlying the formation of bone-voids structures.

## Conclusions

In the present study, implantation of *E. coli*-derived rhBMP-2 combined with BMSCs not only increased the amount of bone formation, but also stabilized the bone mineral density and reduced the non-uniformity. Therefore, the results of the present study suggest that transplantation of osteoprogenitor cells together with rhBMP-2 is important for reconstruction of large bone defects to achieve stable therapeutic outcomes. However, the histological examinations wre performed at only 12 weeks after transplantation. This time point is regarded as the period required for complete main regenerated bone formation, because no significant differences were observed in the trabecular areas among all samples (data not shown). Therefore, it is necessary to perform histological examination at several time point to evaluate t the process underlying the regenerated bone formation. In addition, osteoprogenitor cells such as BMSCs have the disadvantage of increased risk of infection because of the need for in vitro expansion and culture, and also require more complex surgical procedures. *E. coli*-derived rhBMP-2 promotes the differentiation of undifferentiated cells into osteoblasts [28]. This effect may be even larger for osteoprogenitor cell-rich bone marrow and cancellous bone than for BMSCs. Therefore, it may be possible to use other cells and avoid the risk imposed by in vitro culture. In future studies, the combined use of rhBMP-2 with autologous transplantable tissue should be investigated to further improve this technique for clinical application.
